# Does overnight duty affect vascular endothelial function?

**DOI:** 10.1186/s12872-021-02277-y

**Published:** 2021-09-27

**Authors:** A. Garu, Eri Nitta, Yuri Yoshida, Erika Yata, Akari Tsunematsu, Tsuyoshi Araki, Atsushi Nagai, Shozo Yano

**Affiliations:** 1 Department of Neurology, Faculty of Medicine, Shimane University, Shimane, Japan; 2 Department of Clinical Laboratory Medicine, Shimane University Hospital, Shimane, Japan; 3 Department of Laboratory Medicine, Faculty of Medicine, Shimane University, Shimane, Japan

**Keywords:** Reactive hyperemia index (RHI), Peripheral arterial tonometry (PAT), Vascular endothelial function, Fatigue, Sleep

## Abstract

**Background:**

The reactive hyperemia index (RHI), which is obtained from the measurement of peripheral arterial tonometry (PAT), is highly associated with the percentage change in the end-diastolic arterial diameter (%flow-mediated dilatation) at reactive hyperemia. Low RHI is reported to be a mortality risk in patients with a high risk of cardiovascular (CV) disease. CV events are thought to be induced by physical and mental stress, including long-term fatigue and lack of sleep. However, the relationship between fatigue, lack of sleep, and endothelial function has not yet been established.

**Methods:**

Healthy hospital workers (n = 13, 6 men and 7 women) with an average age of 31.6 years were assigned to this study after they provided written informed consent. During the study period, we conducted 72 measurements of reactive hyperemia-peripheral arterial tonometry (RH-PAT) in the morning before or after their duty. At each measurement of the RH-PAT, we recorded the participants’ hours of sleep and evaluated their degree of fatigue using a visual analog scale (VAS).

**Results:**

Although the VAS was significantly less (36 ± 16% and 64 ± 12%, *p* < 0.001) and the hours of sleep were longer (6.0 ± 1.1 h and 2.3 ± 1.0 h, *p* < 0.001) before duty compared to those after duty, the RHI was comparable between them (2.12 ± 0.53 vs. 1.97 ± 0.50, *p* = 0.21). The VAS score was significantly higher in participants with low RHI (< 1.67) than in those with normal RHI (≥ 2.07) (59 ± 13% and 46 ± 21%, respectively, *p* < 0.05). However, binary logistic regression showed no significant association between low RHI and the VAS when adjusted for systemic blood pressure (SBP) and heart rate variability (HRV). In a simple regression analysis, the RHI was significantly correlated with the VAS score but not with sleep duration. A multiple linear regression analysis also showed no significant association between the RHI and VAS scores after adjustment for SBP and HRV.

**Conclusions:**

Vascular endothelial function was not associated with overnight duty, hours of sleep, or degree of fatigue in healthy young adults. Since the RHI may be decreased in severe fatigue conditions through autonomic nerve activity, one should consider the physical and mental conditions of the examinee when evaluating the RH-PAT results.

**Supplementary Information:**

The online version contains supplementary material available at 10.1186/s12872-021-02277-y.

## Background

In recent years, the role of the endothelium in the development of clinical cardiovascular disease (CVD) has been well established [1]. Endothelial dysfunction, characterized by reduced availability of nitric oxide (NO), has been recognized as a clear pathophysiological state that is involved in the development of atherosclerosis and CVD. Endothelial dysfunction is the hallmark of the early stage of coronary artery disease [2]. If present in coronary arteries or peripheral vessels, it is an independent predictor of cardiovascular events. Given that endothelial dysfunction is reversible, early detection of this disorder may have therapeutic and prognostic implications [3]. Therefore, early detection of vascular damage before clinical CVD development has become the goal of prevention [4, 5].

Vascular dysfunction, which may progress to vascular failure, has been evaluated using several methods. Assessment of coronary artery endothelial function, which is regarded as the "gold standard" for endothelial function testing, is invasive because vasodilation and increases in the coronary blood flow should be measured by intravascular infusion of vasoactive agents [6]. In contrast to such invasive procedures, non-invasive techniques have been commonly used, such as flow-mediated dilation (FMD) of the brachial artery and peripheral arterial tonometry (PAT) [7–9]. FMD reflects conduit artery reactivity [10], while PAT represents microvascular status [11]. FMD is technically demanding as it requires specific training, depends on the operator, and the methodology varies across different laboratories [12–14]. Compared with traditional FMD, the advantages of PAT include non-operator-related standardized measurements. Because its analysis is carried out using computer automatic algorithms, there is no difference between observers.

The reactive hyperemia index (RHI), which is obtained from the measurement of PAT, is highly associated with the percentage change in the end-diastolic arterial diameter (%flow-mediated dilatation, %FMD) at reactive hyperemia [11]. A low RHI is reported to be a mortality risk in patients at a high risk of CVD [15]. It has been reported that long-term fatigue or exhaustion is related to CVD [16, 17]. In addition to exhaustion, CVD is also related to a lack of sleep, anxiety, and lack of social support [18–20]. Therefore, fatigue is most likely involved in the pathogenesis of endothelial dysfunction and CVD. However, the relationship between the degree of fatigue after long duration of work and changes in endothelial function has not yet been established. Because fatigue is sometimes associated with lack of sleep, we conducted this study to measure the endothelial function of on-duty workers using RH-PAT and to evaluate the effect of overnight duty, fatigue, or lack of sleep on vascular endothelial function.

## Methods

### Experimental design

The study protocol was conducted in accordance with the Declaration of Helsinki and approved by the ethical committee of the Shimane University Faculty of Medicine (#20191106-1). Written informed consent was obtained from all the participants in this study.

The primary endpoint in this study was the changes in endothelial function after overnight duty. The secondary endpoint was the relationship between endothelial function and fatigue or sleep. The intervention was duty work, where the participants may sleep for some time depending on the work. A total of six RH-PAT examinations of vascular endothelial function for each subject, three inspections before daytime work, and three inspections after nighttime work, were independently performed in the morning before breakfast. To measure the degree of fatigue, the Chalder Fatigue Question (CFQ) scale and the Visual Analog Scale (VAS) were used, and the participants’ sleep duration on the night before the RH-PAT examination was recorded each time.

### Subjects

We recruited healthy volunteers over the age of 20 years who had nighttime duty from Shimane University Hospital as experimental subjects. Thirteen healthy hospital workers (6 men and 7 women) with an average age of 31.6 ± 8.6 years and BMI of 20.8 ± 1.7 kg/m^2^, free from medication and without any former history of CVD, hypertension, or diabetes mellitus were registered to this study.

### Assessment of vascular endothelial function using Endo-PAT

Endothelial function and HRV were estimated using the Endo-PAT device (Itamar Medical Ltd., Caesarea, Israel), which is a non-invasive technology that can record the pulse wave amplitude of the finger arteries with a pneumatic probe to capture pulsatile plethysmograph and detect peripheral arterial tone. Peripheral arterial tonometry (PAT) was performed in response to reactive hyperemia. In the 12 h before the PAT, subjects refrained from smoking, drinking caffeinated beverages, and eating food. In the examination, the subject was in a quiet room with a constant temperature of 20–24 °C and in a comfortable sitting position with their hands at the heart level. A finger probe was placed on the index finger of each hand, and the pressure cuff was placed on the upper arm. The RH protocol included a 7-min baseline measurement, and then the blood pressure cuff on the test arm was inflated to 60 mmHg above the baseline systolic pressure or at least 200 mmHg for 5 min. The occlusion of pulsatile arterial flow was confirmed by the reduction of the PAT tracing to zero. After 5 min, the cuff was deflated, and the PAT tracing was recorded for an additional 5 min. A computer algorithm that automatically normalizes the baseline signal was used to calculate the ratio of the PAT signal to the baseline after the cuff was released and to the contralateral arm. The PAT data were analyzed using a computer in a manner independent of the operator. PAT was also configured to reduce arterial wall tension and increase the range of arterial wall motion without causing potentially confusing vasomotor changes. A value of < 1.67 was considered to indicate endothelial dysfunction, which was determined in other literature in the population at risk of ischemic heart disease [21, 22]. Therefore, in this study, we assigned subjects with RHI < 1.67 to the abnormal group, and those with 1.67 ≤ RHI > 2.07 to the critical value group, and those with RHI ≥ 2.07 to the normal group. The reproducibility of the PAT measurements was verified using statistical procedures reported previously [23]. Endo-PAT provides the augmentation index (AI) and the baseline pulse amplitude. AI (%) adjusted at a heart rate of 75 bpm (AI@75 bpm) was also assessed in this study.

HRV results were available in the time domain and frequency domain formats. The time domain assessed the R-R interval, the standard deviation of normal-to-normal intervals (SDNN), the square root of the mean squared differences of successive normal-to-normal intervals (rMSSD), the number of N–N intervals differing by more than 50 ms (NN50), the NN50 divided by the total number of N–N intervals (pNN50), and the HRV triangular index. The rMSSD reflects short-term heart rate variability, whereas SDNN is attributed to long-term changes in heart rate variability. The SDNN represents the sympathetic and parasympathetic nerve activity, but it does not allow us to distinguish when changes in HRV are due to an increase in sympathetic tone or the withdrawal of vagal tone. The rMSSD and pNN50 indices represent parasympathetic nerve activity. In the frequency domain, data were analyzed for low frequency (LF), high frequency (HF), and the ratio between low-frequency and high-frequency (LF/HF). HF (0.15–0.4 Hz) is related to parasympathetic nerve activity, and LF (0.04–0.15 Hz) is related to sympathetic and parasympathetic nerve activities. The LF/HF ratio represents the sympathetic-vagal balance [24].

### Assessment of the degree of fatigue using CFQ and VAS scale

The CFQ scale was used to assess fatigue. The scale was developed as a brief assessment tool for primary and secondary care. The version we used includes 11 items that consist of questions about fatigue symptoms, such as tiredness, sleepiness, lack of energy, insufficient muscle strength, difficulty in concentration, and memory. Each item required participants to rate the frequency of symptoms by choosing among four options: “less than usual,” “no more than usual,” “more than usual,” and “much more than usual.” A score ranging from 0 to 3 was given with the use of the Likert scale. The total score of the scale was obtained by adding the rating for each item, ranging from 0 to 33. Alternatively, a bimodal score ranging from 0 to 11 can be used where response options “less than usual” and “no more than usual” are given scores of 0, and “more than usual” and “much more than usual” are given scores of 1. Our study defined 0–11 points as less fatigue, 12–22 as moderate fatigue, and ≥ 23 as severe fatigue. The VAS scale was recorded for each examination based on the degree of subjective fatigue.

### Statistical analysis

All data were presented as the mean ± standard deviation (SD), or frequency and percentage. For parametric data with a normal distribution, Student’s *t*-tests for paired observations were used as appropriate. Categorical data were evaluated using ANOVA and Fisher’s least significant difference test. Associations between RHI, sleep, and fatigue were examined using simple linear regression. The association between RHI and VAS scores was examined by multiple linear regression analysis to adjust for SBP, SDNP, rMSSD, and HR. Binary logistic regression models were used to explore the association between the RHI (dependent variable) and variates (independent variables) and expressed as odds ratios (ORs) and 95% confidence intervals (CIs). Statistical significance was set at *p* < 0.05. All statistical analyses were conducted using SPSS (version 25.0; IBM Corp., Armonk, NY, USA).

## Results

### RHI distribution before and after duty

This study was conducted with 13 healthy participants, and the data were collected for statistical analysis. A total of 72 RH-PAT examinations were completed except for six other examinations. The RHI, CFQ, VAS scale, sleep duration, heart rate (HR), and HRV of the study participants were evaluated to analyze the changes in vascular endothelial function before and after duty. The results showed that the RHI was not significantly different between before and after duty (2.12 ± 0.53 and 1.97 ± 0.50, *p* = 0.21) (Table 1).

**Table 1:** Comparison between before duty and after duty

Variables	Before duty	After duty	*p* value
	Mean ± SD	Mean ± SD	
CFQ scale	9 ± 4	17 ± 4	< 0.05
Sleep duration (h)	6.0 ± 1.2	2.3 ± 1.0	< 0.05
VAS scale (%)	36 ± 16	64 ± 12	< 0.05
RHI	2.12 ± 0.53	1.97 ± 0.50	0.21
HR (bpm)	69 ± 9	64 ± 7	0.01
SBP (mmHg)	110 ± 9	112 ± 11	0.35
DBP (mmHg)	64 ± 7	64 ± 7	0.98
SDNN (ms)	46.59 ± 17.27	53.52 ± 15.28	0.08
rMSSD (ms)	40.27 ± 22.37	51.63 ± 23.33	0.04
NN50 (ms)	23.32 ± 22.89	35.06 ± 22.83	0.03
pNN50 (ms)	0.96 ± 3.80	0.12 ± 0.08	0.18
LF (ms^2^)	12.49 ± 4.51	14.16 ± 4.12	0.72
HF (ms^2^)	116.09 ± 59.44	120.92 ± 52.75	0.62
LF/HF	177.97 ± 68.65	186.20 ± 70.11	0.47
AI (%)	0.77 ± 0.54	0.90 ± 0.91	0.33
AI@75 bpm (%)	− 2.54 ± 11.06	0.29 ± 13.18	0.97

Comparison of PAT data of healthy people before and after duty are shown in Table 1. Data are presented here as mean ± standard deviation

AI, augmentation index; AI@75 bpm, augmentation index adjusted at heart rate 75 bpm; CFQ, Chalder Fatigue Question; DBP, diastolic blood pressure; HF, high frequency; HR, heart rate; LF, low frequency; LF/HF, the ratio between low-frequency and high-frequency; N, number; VAS, visual analog scale; NN50, number of N–N differing by more than 50 ms; pNN50, NN50 divided by the total number of N–N intervals; RHI, Reactive hyperemia index; rMSSD, root mean squared standard deviation; SBP, systolic blood pressure; SD, standard deviation; SDNN, standard deviation of normal-to-normal intervals

Statistical analysis was done with paired *t* test, where *p* < 0.05 was considered as significant

The CFQ scale significantly increased after duty compared with before duty. A similar change in the VAS scores was also observed (Table 1). A significant reduction in sleep duration and HR was found after duty, while the values of rMSSD and NN50 were significantly increased (Table 1).

These results suggest that duty affects the subjective feeling of fatigue and the autonomic nervous system, but not vascular endothelial function. Since some participants felt fatigued even before duty for unknown reasons, we focused on the relationship between the degree of fatigue and vascular endothelial function.

### Association between fatigue, sleep duration, HRV, and endothelial function

To investigate whether fatigue or sleep deprivation may affect vascular endothelial function, we performed further analyses. Participants were divided into three groups according to RHI: normal, critical value, and abnormal group. Although ANOVA and posthoc analysis indicated no significant difference in sleep duration or CFQ scale among them, the VAS was significantly higher in the abnormal RHI group than in the normal RHI group (59 ± 13% and 46 ± 21%, respectively, *p* < 0.05) (Table 2). This suggests that a high degree of fatigue monitored by the VAS scale, but not the CFQ scale, may be associated with vascular endothelial dysfunction.

**Table 2:** Comparison of sleep, fatigue HRV and AI among RHI three groups

Variables	RHI	N	Mean	SD	*p* value
Sleep duration (h)	Normal	34	4.4	2.2	–
	Critical value	20	4.2	2.4	0.84
	Abnormal	18	4.0	1.9	0.61
VAS scale (%)	Normal	34	46	21	–
	Critical value	20	48	21	0.65
	Abnormal	18	59	13	0.02
CFQ scale	Normal	34	13	6	–
	Critical value	20	12	7	0.35
	Abnormal	18	14	5	0.74
SBP	Normal	34	108	9	–
	Critical value	20	112	10	0.19
	Abnormal	18	114	11	0.07
DBP	Normal	34	64	8	–
	Critical value	20	66	7	0.23
	Abnormal	18	63	5	0.85
HR	Normal	34	68	9	–
	Critical value	20	65	8	0.17
	Abnormal	18	64	6	0.09
SDNN (ms)	Normal	34	46.05	16.14	–
	Critical value	20	52.39	12.41	0.18
	Abnormal	18	54.22	20.41	0.09
rMSSD (ms)	Normal	34	40.84	19.82	–
	Critical value	20	46.49	16.74	0.39
	Abnormal	18	53.77	32.91	0.06
NN50 (ms)	Normal	34	24.74	23.26	–
	Critical value	20	33.00	23.76	0.22
	Abnormal	18	32.72	23.39	0.25
pNN50 (ms)	Normal	34	55.71	280.91	–
	Critical value	20	10.84	8.17	0.57
	Abnormal	18	103.60	395.24	0.55
LF (ms^2^)	Normal	34	109.69	64.08	–
	Critical value	20	138.01	52.48	0.07
	Abnormal	18	113.23	37.31	0.83
HF (ms^2^)	Normal	34	180.30	77.14	–
	Critical value	20	187.03	72.97	0.73
	Abnormal	18	179.49	48.35	0.97
LF/HF	Normal	34	0.76	0.62	–
	Critical value	20	1.03	0.94	0.20
	Abnormal	18	0.76	0.70	0.99
AI (%)	Mild	33	− 3.00	11.53	–
	moderate	35	0.68	11.83	0.22
	Severe	5	− 1.60	18.43	0.81
AI@75 bpm (%)	Normal	34	− 5.32	10.24	−
	Critical value	20	− 5.20	14.20	0.97
	Abnormal	18	− 10.83	9.19	0.10

Table shows the correlation between different RHI groups and HRV, sleep, and fatigue. Participants were divided into 3 groups by RHI: normal (< 1.67), critical value (1.67–2.07), and abnormal (≥ 2.07) groups. Data are presented here as mean ± standard deviation

AI, augmentation index; AI@75 bpm, augmentation index adjusted at heart rate 75 bpm; CFQ, Chalder Fatigue Question; DBP, diastolic blood pressure; HF, high frequency; HR, heart rate; HRV, heart rate variability; LF, low frequency; LF/HF, the ratio between low-frequency and high-frequency; N, number; VAS, visual analog scale; NN50, number of N–N differing by more than 50 ms; pNN50, NN50 divided by the total number of N–N intervals; RHI, Reactive hyperemia index; rMSSD, root mean squared standard deviation; SBP, systolic blood pressure; SD, standard deviation; SDNN, standard deviation of normal-to-normal intervals

Statistical analysis was done with ANOVA test and Fisher's LSD test, where *p* < 0.05 was considered as significant

Compared with the normal RHI group, SBP, SDNN, and rMSSD tended to increase in the abnormal group, and the LF of the critical value group tended to increase, whereas the HR of the abnormal group tended to decrease (Table 2). Thus, we performed a binary logistic regression analysis for low RHI to adjust for SBP, SDNN, and rMSSD (Table 3). The results showed that the VAS score was independently associated with RHI in model 1 (OR 1.04 [95%CI 1.00–1.07]; *p* = 0.026) and remained independently associated with RHI when SBP was adjusted in model 2 (OR 1.03 [95%CI 1.00–1.07]; *p* = 0.044); however, no statistical significance was observed when SBP and HRV were adjusted in model 3 (OR 1.03 [95%CI 1.00–1.07]; *p* = 0.056) (Table 3).

**Table 3:** Odds ratios in three models of binary logistic regression analysis for low RHI

Variables	OR (95%CI)	*p* value
Model 1		
VAS scale (%)	1.04 (1.00–1.07)	0.026
Model 2		
VAS scale (%)	1.03 (1.00–1.07)	0.044
SBP (mmHg)	1.03 (0.97–1.09)	0.303
Model 3		
VAS scale (%)	1.03 (1.00–1.07)	0.056
SBP (mmHg)	1.04 (0.97–1.10)	0.267
rMSSD (ms)	1.02 (0.97–1.06)	0.431
SDNN (ms)	0.99 (0.93–1.06)	0.785
HR (bpm)	0.97 (0.89–1.06)	0.501

Table shows the participants were divided into 2 groups by RHI: normal (< 1.67), and abnormal (≥ 1.67) groups

CI, confidence interval; HR, heart rate; OR, odds ratio; RHI, Reactive hyperemia index; rMSSD, root mean squared standard deviation; SBP, systolic blood pressure; SDNN, standard deviation of normal-to-normal intervals; VAS, visual analog scale

Statistical analysis was done with binary logistic regression analysis where *p* < 0.05 was considered as significant

**Fig. 1:**
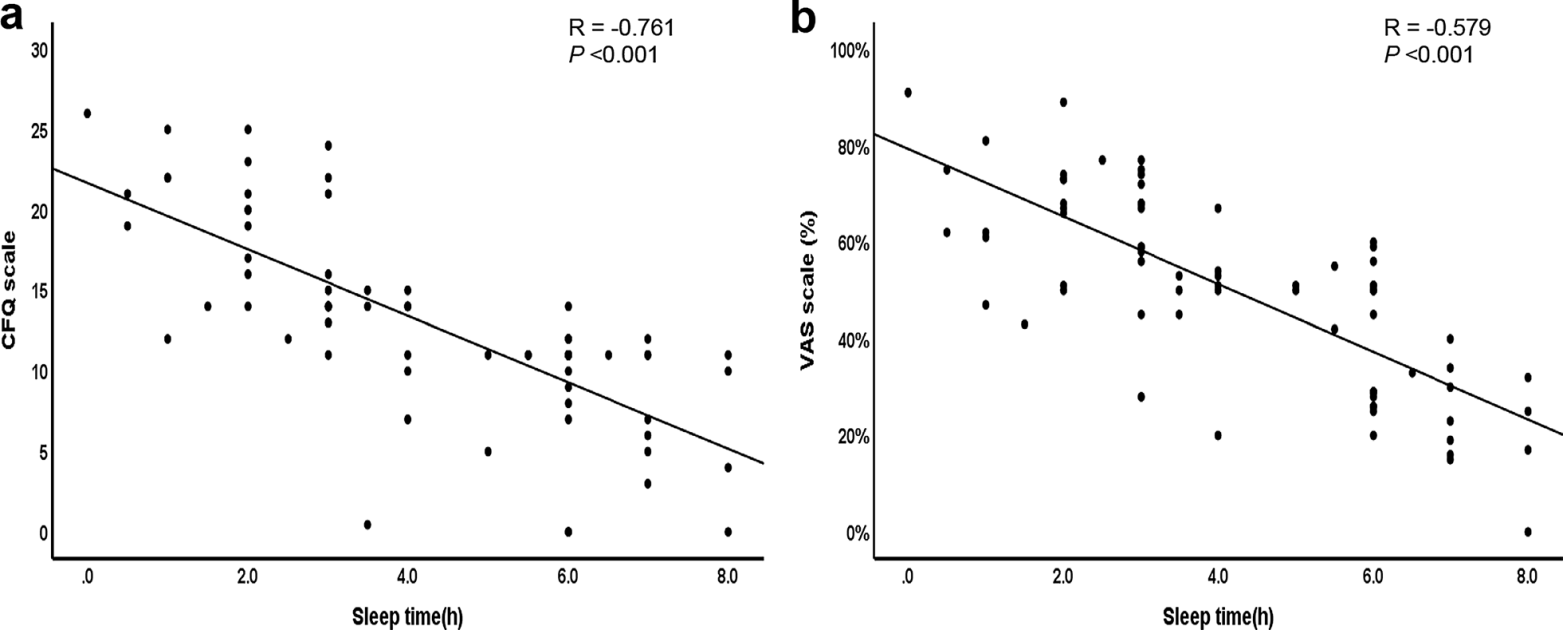
Relationship between the degree of fatigue and sleep duration in 72 RH-PAT examinations. There was a significant association between sleep and the CFQ scale (**a**) or VAS scale (**b**). Statistical significance was set at *p* < 0.05

**Fig. 2:**
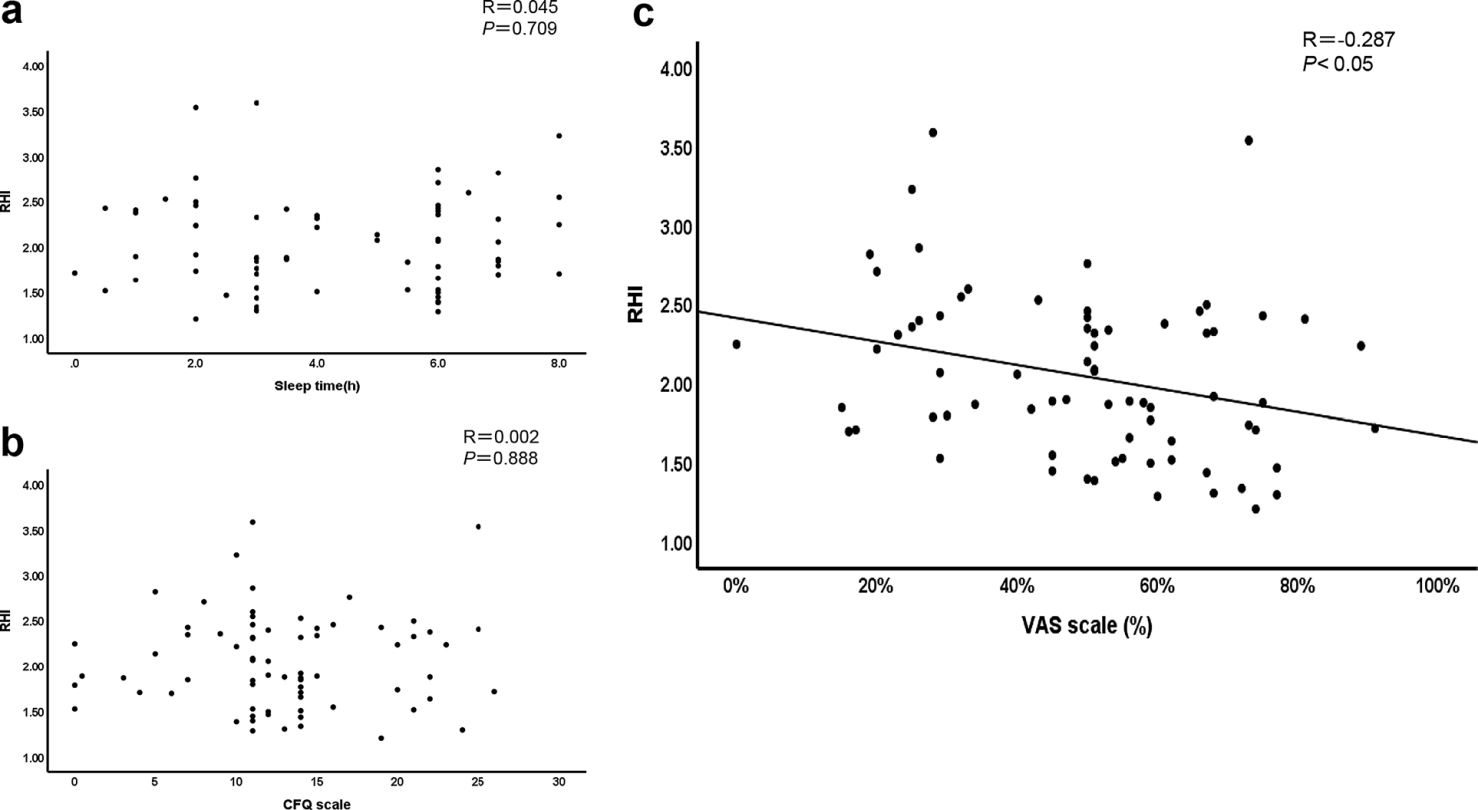
Relationship between RHI and sleep duration or the degree of fatigue in 72 RH-PAT examinations. No significant association was observed between the reactive hyperemia index (RHI) and sleep duration (**a**) or CFQ scale (**b**) in 13 healthy subjects. However, a significant correlation was observed between the RHI and VAS scale (**c**). A linear regression line is shown (*p* < 0.05, considered significant)

In a simple regression analysis, an inverse association between sleep duration, CFQ scale (r = − 0.761, *p* < 0.05), and VAS (r = − 0.579, *p* < 0.05) were observed (Fig. 1), suggesting that the degree of fatigue is relatively parallel with lack of sleep. As for endothelial function, no significant correlation was found between the RHI and sleep duration (r = 0.045, *p* = 0.71) (Fig. 2a) or the CFQ scale (r = 0.002, *p* = 0.89) (Fig. 2b). In contrast, the RHI was significantly correlated with the VAS score (r = − 0.287, *p* < 0.05) (Fig. 2c). Furthermore, no association was found between AI@75 bpm and sleep or fatigue (Additional file 1: Supplementary Figure S1). In a multiple linear regression analysis, although the RHI was significantly correlated with the VAS even after adjusting for SBP, no significant association was found when SDNN, rMSSD, and HR were added for the adjustment (Table 4). RHI was negatively correlated with SBP and positively correlated with HR. These results are consistent with those from binary logistic regression analyses.

**Table 4:** A multiple linear regression analysis for RHI

Variables	Beta	SE	Standard beta	*p* value	VIF
Model 1					
SBP (mmHg)	− 0.01	0.01	− 0.21	0.071	1.04
VAS scale (%)	− 0.01	0.00	− 0.25	0.034	1.04
Model 2					
SBP (mmHg)	− 0.01	0.01	− 0.26	0.026	1.07
SDNN (ms)	− 0.01	0.01	− 0.16	0.371	2.63
rMSSD (ms)	0.00	0.00	0.07	0.703	2.80
HR (bpm)	0.02	0.01	0.30	0.017	1.19
VAS scale (%)	− 0.01	0.00	− 0.19	0.100	1.10

Table shows the participants were divided into 2 groups by RHI: normal (< 1.67), and abnormal (≥ 1.67) groups

HR, heart rate; RHI, Reactive hyperemia index; rMSSD, root mean squared standard deviation; SBP, systolic blood pressure; SDNN, standard deviation of normal-to-normal intervals; SE, standard error; VAS, visual analog scale; VIF, variance inflation factor

Statistical analysis was done with multiple linear regression analysis where *p* < 0.05 was considered as significant

## Discussion

Endothelial dysfunction is considered an initial step in the pathophysiology of atherosclerosis and cardiovascular complications [25]. However, whether overnight duty affects vascular endothelial function, and if so, what is the mechanism remain unclear. In this study, we focused on the relation to fatigue and found that compared with before duty, both CFQ and VAS scores increased after duty without significant differences in RHI, suggesting that overnight duty induced the subjective feeling fatigue and not endothelial dysfunction. We also found that the RHI was significantly correlated with the VAS score after adjusting for SBP but not after adjusting for HRV. This suggests that low RHI results may be associated with severe fatigue conditions, most likely through sympathetic and/or parasympathetic nerve activity.

The present study showed that HR after duty was significantly reduced compared to that before duty. In contrast, rMSSD and NN50 were significantly increased, which is consistent with previous findings [26]. The interaction can occur intensively through the influence of endothelial mediator, mainly NO, on the function of the autonomic nervous system. Nitric oxide synthases, including endothelial and neuronal isoforms, are distributed in the central nervous system and are responsible for autonomous regulation [27, 28]. Human studies have also shown that NO may enhance cardiac vagal nerve control and inhibit sympathetic nerve activity [29, 30]. In Tables 3 and 4, we found that the correction by HRV diminished the statistical significance of the association between RHI and VAS, suggesting that severe fatigue conditions may lead to low RHI results through autonomic nerve activity.

We conducted multiple linear regression analyses and found that SBP was negatively correlated with RHI, which is the same as in previous studies [31]. Endothelial dysfunction promotes apoptosis of vascular endothelial cells [32], leading to arteriosclerosis [33], thereby reducing the ability of the vascular system to buffer the rise in SBP caused by left ventricular ejection [34]. Endothelial dysfunction is characterized by decreased bioavailability of vasodilators, especially NO, and/or increased vasoconstrictors such as endothelin [35]. The resulting imbalance between vasodilation and vasoconstriction may cause damage to endothelium-dependent vasodilation [36].

In simple linear regression, we clearly showed an inverse association between the degree of fatigue and sleep duration. Although we did not find a significant relationship between sleep duration and endothelial function in healthy young workers, previous short-term physiological studies have shown that insufficient and/or inadequate sleep is associated with slowed glucose metabolism and an increase in inflammatory mediators, suggesting that lack of sleep is sufficient to trigger the development of endothelial dysfunction [37]. Takase et al. observed that a combination of 4-week stress and sleep deprivation attenuated endothelial function measured by FMD in the brachial artery in 30 healthy college students [38]. It is reasonable to hypothesize that long-term lack of sleep may contribute to stress-induced sympathetic or parasympathetic nerve activity changes, hormonal changes, and increase in basal levels of inflammatory mediators and coagulation factors, thereby triggering CVD development even in healthy people. This is an important issue in public health and health risk management and deserves further attention.

Endothelial function may be a functional expression of the overall burden of cardiovascular risk factors, which reflects the sum of all vascular protective factors and becomes a parameter of disease activity [39]. According to epidemiological evidence, fatigue and/or mental stress are related to CVD development [16, 17, 40, 41], and the physiological process of fatigue must affect vascular function. We showed that subjectively severe fatigue was related to low RHI without HRV correction, which is consistent of previous studies [42]. However, they measured the RHI before work in the morning and after work in the evening. Another study showed that the FMD at 8:00 and 12:00 was significantly lower than that at 17:00, indicating that morning attenuation of endothelial function should be considered in clinical research and may play an important role in the circadian variation in the occurrence of cardiovascular events [43]. Thus, a morning fasting examination is desirable for measuring endothelial function. Therefore, for all examinations, we measured the RHI in the morning before duty as well as after overnight duty.

Endo-PAT provides AI, baseline pulse amplitude, and RHI, which correlate with endothelial function and cardiovascular risk factors [8, 44]. The AI is derived from the arterial pulse waveform, which is determined by the ratio between the augmentation pressure and pulse pressure, which is a measure of peripheral arterial stiffness and macrovascular disease. Studies have found that AI is a predictor of adverse cardiovascular events in different patient populations, with higher AI indicating increased end-organ damage [45]. In the present study, however, the participants were all healthy, and the AI was not related to duty, fatigue, or sleep.

Our study has some limitations. First, we had a small number of subjects, which is a major weakness of this study and may result in the inconsistent findings with the majority of other previous works [46–50]; Second, in the present study, we did not consider the quality of sleep and insomnia, which affect vascular endothelial function, respectively [51, 52], where noise-induced impairment of sleep quality has been shown to cause endothelial dysfunction as measured by FMD [53, 54]; and third, fatigue assessment is subjective but not objective. Regarding the discrepancy of our results and other previous works showing overnight duty-associated endothelial dysfunction, small number of our participants and their young and healthy characteristics might be involved. Because sleep disturbance, poor quality of sleep, and insomnia probably induces fatigue feeling, extensive future study is necessary to clarify the mechanism of vascular endothelial dysfunction. Nonetheless, we evaluated the risks other than the non-traditional risk factors, including fatigue and lack of sleep, thus laying a foundation for rational work arrangements and self-health management in our daily lives.

## Conclusions

Vascular endothelial function was not associated with overnight duty, hours of sleep, or degree of fatigue in healthy young adults. Since the RHI may be decreased in severe fatigue conditions through autonomic nerve activity, one should consider the physical and mental conditions of the examinee when evaluating the RH-PAT results.

## Supplementary Information

**Additional file 1. Figure S1: **Relationship between AI adjusted by heart rate at 75bpm (AI@75bpm) and sleep or fatigue. No significant association was observed between AI@75bpm and sleep duration (a), CFQ scale (b), or VAS scale (c).

## Data Availability

The datasets used and/or analyzed during the current study are available from the corresponding author on reasonable request.

## Abbreviations

AI: Augmentation index
CFQ: Chalder fatigue question
CVD: Cardiovascular disease
DBP: Diastolic blood pressure
FMD: Flow-mediated dilatation
HR: Heart rate
HRV: Heart rate variability
NO: Nitric oxide
PAT: Peripheral arterial tonometry
RHI: Reactive hyperemia index
RH-PAT: Reactive hyperemia- peripheral arterial tonometry
SBP: Systolic blood pressure
VAS: Visual analog scale

## References

[1] Widlansky ME, Gokce N, Keaney JF, Vita JA (2003). The clinical implications of endothelial dysfunction. J Am Coll Cardiol.

[2] Ross R (1993). The pathogenesis of atherosclerosis: a perspective for the 1990s. Nature.

[3] Bonetti PO, Lerman LO, Lerman A (2003). Endothelial dysfunction: a marker of atherosclerotic risk. Arterioscler Thromb Vasc Biol.

[4] Yeboah J, Folsom AR, Burke GL, Johnson C, Polak JF, Post W, Lima JA, Crouse JR, Herrington DM (2009). Predictive value of brachial flow-mediated dilation for incident cardiovascular events in a population-based study: the multi-ethnic study of atherosclerosis. Circulation.

[5] Lind L, Berglund L, Larsson A, Sundström J (2011). Endothelial function in resistance and conduit arteries and 5-year risk of cardiovascular disease. Circulation.

[6] Tanaka A, Tomiyama H, Maruhashi T, Matsuzawa Y, Miyoshi T, Kabutoya T, Kario K, Sugiyama S, Munakata M, Ito H (2018). Physiological diagnostic criteria for vascular failure. Hypertension.

[7] Benjamin EJ, Larson MG, Keyes MJ, Mitchell GF, Vasan RS, Keaney JF, Lehman BT, Fan S, Osypiuk E, Vita JA (2004). Clinical correlates and heritability of flow-mediated dilation in the community: the Framingham Heart Study. Circulation.

[8] Hamburg NM, Keyes MJ, Larson MG, Vasan RS, Schnabel R, Pryde MM, Mitchell GF, Sheffy J, Vita JA, Benjamin EJ (2008). Cross-sectional relations of digital vascular function to cardiovascular risk factors in the Framingham Heart Study. Circulation.

[9] Schnabel RB, Schulz A, Wild PS, Sinning CR, Wilde S, Eleftheriadis M, Herkenhoff S, Zeller T, Lubos E, Lackner KJ (2011). Noninvasive vascular function measurement in the community: cross-sectional relations and comparison of methods. Circ Cardiovasc Imaging.

[10] Laurent S, Lacolley P, Brunel P, Laloux B, Pannier B, Safar M (1990). Flow-dependent vasodilation of brachial artery in essential hypertension. Am J Physiol.

[11] Kuvin JT, Patel AR, Sliney KA, Pandian NG, Sheffy J, Schnall RP, Karas RH, Udelson JE (2003). Assessment of peripheral vascular endothelial function with finger arterial pulse wave amplitude. Am Heart J.

[12] Rasmussen JG, Eschen RB, Aardestrup IV, Dethlefsen C, Griffin BA, Schmidt EB (2009). Flow-mediated vasodilatation: variation and interrelationships with plasma lipids and lipoproteins. Scand J Clin Lab Invest.

[13] De Roos NM, Bots ML, Schouten EG, Katan MB (2003). Within-subject variability of flow-mediated vasodilation of the brachial artery in healthy men and women: implications for experimental studies. Ultrasound Med Biol.

[14] Sejda T (2005). R: limitations of non-invasive endothelial function assessment by brachial artery flow-mediated dilatation. Clin Physiol Funct Imaging.

[15] Matsuzawa Y, Sugiyama S, Sumida H, Sugamura K, Nozaki T, Ohba K, Matsubara J, Kurokawa H, Fujisue K, Konishi M (2013). Peripheral endothelial function and cardiovascular events in high-risk patients. J Am Heart Assoc.

[16] Kop WJ, Appels AP (1994). Vital exhaustion predicts new cardiac events after successful coronary angioplasty. Psychosom Med.

[17] Naess H, Nyland HI, Thomassen L, Aarseth J, Myhr KM (2005). Fatigue at long-term follow-up in young adults with cerebral infarction. Cerebrovasc Dis.

[18] Naess H, Waje-Andreassen U, Thomassen L, Nyland H, Myhr KM (2006). Health-related quality of life among young adults with ischemic stroke on long-term follow-up. Stroke.

[19] Alboni P, Favaron E, Paparella N, Sciammarella M, Pedaci M (2008). Is there an association between depression and cardiovascular mortality or sudden death?. J Cardiovasc Med (Hagerstown).

[20] Ketterer MW, Knysz W, Keteyian SJ, Schairer J, Jafri S, Alam M, Farha AJ, Deveshwar S (2008). Cardiovascular symptoms in coronary-artery disease patients are strongly correlated with emotional distress. Psychosomatics.

[21] Yeo TW, Lampah DA, Gitawati R, Tjitra E, Kenangalem E, McNeil YR, Darcy CJ, Granger DL, Weinberg JB, Lopansri BK (2007). Impaired nitric oxide bioavailability and L-arginine reversible endothelial dysfunction in adults with falciparum malaria. J Exp Med.

[22] Yinon D, Lowenstein L, Suraya S, Beloosesky R, Zmora O, Malhotra A, Pillar G (2006). Pre-eclampsia is associated with sleep-disordered breathing and endothelial dysfunction. Eur Respir J.

[23] Bonetti PO, Barsness GW, Keelan PC, Schnell TI, Pumper GM, Kuvin JT, Schnall RP, Holmes DR, Higano ST, Lerman A (2003). Enhanced external counterpulsation improves endothelial function in patients with symptomatic coronary artery disease. J Am Coll Cardiol.

[24] Farinatti P, Neto SR, Dias I, Cunha FA, Bouskela E, Kraemer-Aguiar LG (2016). Short-term resistance training attenuates cardiac autonomic dysfunction in obese adolescents. Pediatr Exerc Sci.

[25] Hasdai D, Lerman A (1999). The assessment of endothelial function in the cardiac catheterization laboratory in patients with risk factors for atherosclerotic coronary artery disease. Herz.

[26] Pinter A, Horvath T, Sarkozi A, Kollai M (2012). Relationship between heart rate variability and endothelial function in healthy subjects. Auton Neurosci.

[27] Carlson SH, Wyss JM (2008). Neurohormonal regulation of the sympathetic nervous system: new insights into central mechanisms of action. Curr Hypertens Rep.

[28] Chowdhary S, Townend JN (2001). Nitric oxide and hypertension: not just an endothelium derived relaxing factor!. J Hum Hypertens.

[29] Chowdhary S, Vaile JC, Fletcher J, Ross HF, Coote JH, Townend JN (2000). Nitric oxide and cardiac autonomic control in humans. Hypertension.

[30] Young CN, Fisher JP, Gallagher KM, Whaley-Connell A, Chaudhary K, Victor RG, Thomas GD, Fadel PJ (2009). Inhibition of nitric oxide synthase evokes central sympatho-excitation in healthy humans. J Physiol.

[31] Yufu K, Takahashi N, Okada N, Shinohara T, Hara M, Saikawa T, Yoshimatsu H (2009). Influence of systolic blood pressure and cigarette smoking on endothelial function in young healthy people. Circ J.

[32] Paterick TE, Fletcher GF (2001). Endothelial function and cardiovascular prevention: role of blood lipids, exercise, and other risk factors. Cardiol Rev.

[33] Mackey RH, Sutton-Tyrrell K, Vaitkevicius PV, Sakkinen PA, Lyles MF, Spurgeon HA, Lakatta EG, Kuller LH (2002). Correlates of aortic stiffness in elderly individuals: a subgroup of the cardiovascular Health Study. Am J Hypertens.

[34] Stewart KJ, Sung J, Silber HA, Fleg JL, Kelemen MD, Turner KL, Bacher AC, Dobrosielski DA, DeRegis JR, Shapiro EP, Ouyang P (2004). Exaggerated exercise blood pressure is related to impaired endothelial vasodilator function. Am J Hypertens.

[35] Lerman A, Burnett JC (1992). Intact and altered endothelium in regulation of vasomotion. Circulation.

[36] Hadi HA, Carr CS, Al Suwaidi J (2005). Endothelial dysfunction: cardiovascular risk factors, therapy, and outcome. Vasc Health Risk Manag.

[37] Mullington JM, Haack M, Toth M, Serrador JM, Meier-Ewert HK (2009). Cardiovascular, inflammatory, and metabolic consequences of sleep deprivation. Prog Cardiovasc Dis.

[38] Takase B, Akima T, Uehata A, Ohsuzu F, Kurita A (2004). Effect of chronic stress and sleep deprivation on both flow-mediated dilation in the brachial artery and the intracellular magnesium level in humans. Clin Cardiol.

[39] Reriani MK, Flammer AJ, Jama A, Lerman LO, Lerman A (2012). Novel functional risk factors for the prediction of cardiovascular events in vulnerable patients following acute coronary syndrome. Circ J.

[40] Melamed S, Shirom A, Toker S, Berliner S, Shapira I (2006). Burnout and risk of cardiovascular disease: evidence, possible causal paths, and promising research directions. Psychol Bull.

[41] Soufer R, Jain H, Yoon AJ (2009). Heart-brain interactions in mental stress-induced myocardial ischemia. Curr Cardiol Rep.

[42] Ohno Y, Hashiguchi T, Maenosono R, Yamashita H, Taira Y, Minowa K, Yamashita Y, Kato Y (2010). Kawahara K-i, Maruyama I: The diagnostic value of endothelial function as a potential sensor of fatigue in health. Vasc Health Risk Manag.

[43] Etsuda H, Takase B, Uehata A, Kusano H, Hamabe A, Kuhara R, Akima T, Matsushima Y, Arakawa K, Satomura K, Kurita A, Ohsuzu F (1999). Morning attenuation of endothelium-dependent, flow-mediated dilation in healthy young men: possible connection to morning peak of cardiac events?. Clin Cardiol.

[44] Bonetti PO, Pumper GM, Higano ST, Holmes DR, Kuvin JT, Lerman A (2004). Noninvasive identification of patients with early coronary atherosclerosis by assessment of digital reactive hyperemia. J Am Coll Cardiol.

[45] Janner JH, Godtfredsen NS, Ladelund S, Vestbo J, Prescott E (2013). High aortic augmentation index predicts mortality and cardiovascular events in men from a general population, but not in women. Eur J Prev Cardiol.

[46] Amir O, Alroy S, Schliamser JE, Asmir I, Shiran A, Flugelman MY, Halon DA, Lewis BS (2004). Brachial artery endothelial function in residents and fellows working night shifts. Am J Cardiol.

[47] Tarzia P, Milo M, Di Franco A, Di Monaco A, Cosenza A, Laurito M, Lanza GA, Crea F (2012). Effect of shift work on endothelial function in young cardiology trainees. Eur J Prev Cardiol.

[48] Wehrens SM, Hampton SM, Skene DJ: Heart rate variability and endothelial function after sleep deprivation and recovery sleep among male shift and non-shift workers. Scand J Work Environ Health 2012:171–181. 10.5271/sjweh.3197 21953310

[49] Charles LE, Zhao S, Fekedulegn D, Violanti JM, Andrew ME, Burchfiel CM (2016). Shiftwork and decline in endothelial function among police officers. Am J Ind Med.

[50] Shimada K, Fukuda S, Maeda K, Kawasaki T, Kono Y, Jissho S, Taguchi H, Yoshiyama M, Yoshikawa J (2011). Aromatherapy alleviates endothelial dysfunction of medical staff after night-shift work: preliminary observations. Hypertens Res.

[51] Cooper DC, Ziegler MG, Milic MS, Ancoli-Israel S, Mills PJ, Loredo JS, Von Kanel R, Dimsdale JE (2014). Endothelial function and sleep: associations of flow-mediated dilation with perceived sleep quality and rapid eye movement (REM) sleep. J Sleep Res.

[52] Routledge FS, Dunbar SB, Higgins M, Rogers AE, Feeley C, Ioachimescu O, Euwer K, Eapen D, Quyyumi A (2017). Insomnia symptoms are associated with abnormal endothelial function. J Cardiovasc Nurs.

[53] Herzog J, Schmidt FP, Hahad O, Mahmoudpour SH, Mangold AK, Andreo PG, Prochaska J, Koeck T, Wild PS, Sørensen M (2019). Acute exposure to nocturnal train noise induces endothelial dysfunction and pro-thromboinflammatory changes of the plasma proteome in healthy subjects. Basic Res Cardiol.

[54] Schmidt FP, Herzog J, Schnorbus B, Ostad MA, Lasetzki L, Hahad O, Schafers G, Gori T, Sorensen M, Daiber A (2021). The impact of aircraft noise on vascular and cardiac function in relation to noise event number: a randomized trial. Cardiovasc Res.

