# Supplementary figures and images for: Does overnight duty affect vascular endothelial function?
Source: BMC Cardiovasc Disord. 2021 Sep 27;21:467. doi: 10.1186/s12872-021-02277-y (PMC8474775; doi:10.1186/s12872-021-02277-y)

## Slide 1
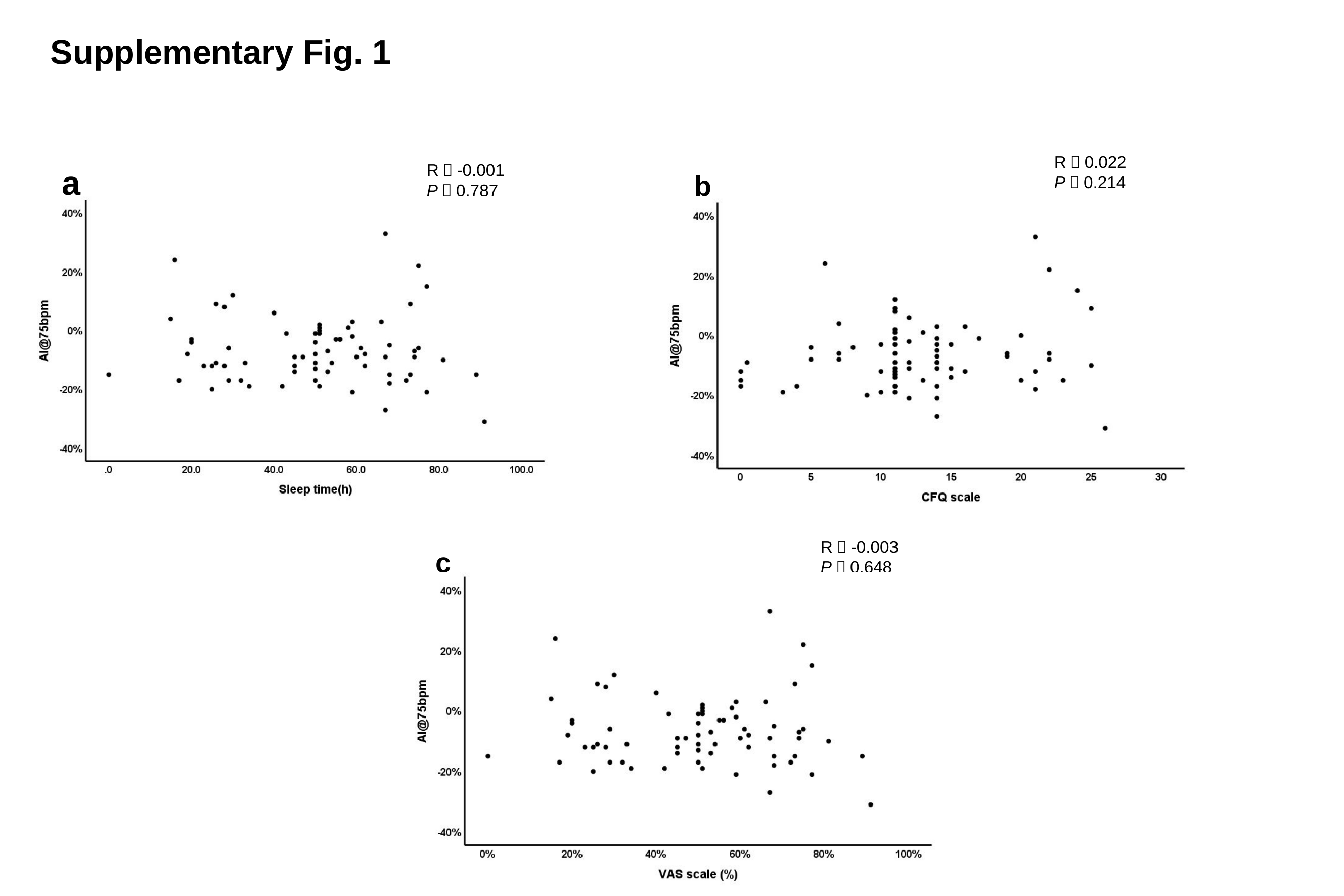

Supplementary Fig. 1
R＝0.022
P＝0.214
R＝-0.001
P＝0.787
a
b
R＝-0.003
P＝0.648
c

Supplement: Supplementary file 1 — Additional file 1. Figure S1: Relationship between AI adjusted by heart rate at 75bpm (AI@75bpm) and sleep or fatigue. No significant association was observed between AI@75bpm and sleep duration (a), CFQ scale (b), or VAS scale (c). [file 12872_2021_2277_MOESM1_ESM.pptx]
